# Gravity-Based Flow Efficient Perfusion Culture System for Spheroids Mimicking Liver Inflammation

**DOI:** 10.3390/biomedicines9101369

**Published:** 2021-10-01

**Authors:** Young-Su Kim, Arun Asif, Abdul Rahim Chethikkattuveli Salih, Jae-Wook Lee, Ki-Nam Hyun, Kyung-Hyun Choi

**Affiliations:** 1 BioSpero Inc., Jeju-si 63243, Jeju-do, Korea; youngsu1742@naver.com (Y.-S.K.); gusrlska8204@naver.com (K.-N.H.); 2 Advanced Micro Mechatronics Lab, Department of Mechatronics Engineering, Jeju National University, Jeju-si 63243, Jeju-do, Korea; arunasif@hotmail.com (A.A.); abdul.rahim350@gmail.com (A.R.C.S.); jaewook482@gmail.com (J.-W.L.)

**Keywords:** microfluidics, liver, 3D culture, spheroids, hepatoxicity, inflammation

## Abstract

The spheroid culture system provides an efficient method to emulate organ-specific pathophysiology, overcoming the traditional two-dimensional (2D) cell culture limitations. The intervention of microfluidics in the spheroid culture platform has the potential to enhance the capacity of in vitro microphysiological tissues for disease modeling. Conventionally, spheroid culture is carried out in static conditions, making the media nutrient-deficient around the spheroid periphery. The current approach tries to enhance the capacity of the spheroid culture platform by integrating the perfusion channel for dynamic culture conditions. A pro-inflammatory hepatic model was emulated using a coculture of HepG2 cell line, fibroblasts, and endothelial cells for validating the spheroid culture plate with a perfusable channel across the spheroid well. Enhanced proliferation and metabolic capacity of the microphysiological model were observed and further validated by metabolic assays. A comparative analysis of static and dynamic conditions validated the advantage of spheroid culture with dynamic media flow. Hepatic spheroids were found to have improved proliferation in dynamic flow conditions as compared to the static culture platform. The perfusable culture system for spheroids is more physiologically relevant as compared to the static spheroid culture system for disease and drug analysis.

## 1. Introduction

Spheroid culture systems provide the opportunity to formulate sophisticated three-dimensional (3D) tissues to mimic the organ-specific microenvironment and pathophysiology. The level of relevance of a microphysiological system (MPS) with human physiology makes them ideal for identifying the novel therapeutics. The organ-on-a-chip and spheroid culture systems are evolving to better mimic human pathophysiology, which is being enhanced by the integration of sensors and robotics [1,2,3,4,5,6,7,8]. Among MPS, spheroids are emerging with the potential to mimic human tissues in a 3D shape and function [9,10,11].

Spheroids are generated using multiple methods including culture on ultra-low attachment (ULA) surfaces, bioreactors [12], and cell aggregation in hanging drops [13]. Three-dimensional (3D) spheroid models have also been considered in assay- and automation-ready plate formats, including 96-well, 384-well, and 1536-well plates [14,15]. Creating a non-adherent surface averts the attachment of cells to the substrate derives spheroid development. Traditionally, liquid overlay techniques have been used to establish a non-adherent surface for cells to form 3D spheres, which include poly-2-hydroxyethyl methacrylate (poly-HEMA), pluronic acid, or 1–2% agarose coating on the substrate surface [16]. Mainly, spheroid formation is achieved in 96-well plates or agarose microwell consisting of non-adherent bottoms [17]. Another accomplished method consists of the hanging drop technique that utilizes microfluidic devices that are slightly complex but provide more control of spheroid size and composition. The hanging drop technique is considerably simple for having consistent spheroid sizes yet the major drawback is lower throughput [18]. Spheroids present in a non-microfluidic static culture systems tend to have a limited media exposure across the surfaces of the spheroid, making them nutrient-deficient with a simultaneous waste accumulation [19].

Conversely, microfluidic devices have been progressively used since they help in controlling the spheroid formation [20]. The microfluidic system also facilitates the formulation, long-term maintenance, and analysis of spheroids inside a single device [21]. The hepatic spheroid culture approach presents a reliable alternative to mimic physiologically relevant drug and disease analysis [22]. As the liver is the metabolic hub for disease and drug responses, emulating hepatic physiology in spheroid culture systems can potentially enhance the predictive capacity of in vitro liver models [23]. The rise of reactive oxygen species in a hepatic microenvironment in response to disease and drugs causes cellular stress [24]. Continuous evidence has suggested that oxidative stress leads to the buildup of hepatic inflammation [25]. One of the major biomarkers for the pro-inflammatory environment is IL-1β, which contributes to oxidative stress, subsequently leading to inflammation, which further develops into hepatocellular carcinoma [26,27,28,29]. Furthermore, continuous evidence has supported the higher expression of tumor necrosis factor alpha (TNF-α) and interleukin 6 (IL-6) to monitor liver inflammation [30,31,32,33]. Previously studied liver tissue models in the microfluidic spheroid culture system lack endothelial cells and a culture platform with microfluidic flow capacity. By improving the biomarker profiles and growth characteristics, the microfluidic perfusion of media enhances the overall spheroid culture system.

The current study proposes a plate-based microfluidic spheroid culture method to support a dynamic media flow. The spheroid plate was designed to hold 24-wells with perfusion channels. The validation of spheroid function was compared with dynamic and static conditions. A mixed tri-culture of hepatocytes, endothelial cells, and fibroblasts was performed to generate spheroids to assess the liver tissue development with more human relevance. Multiple cell concentrations were analyzed to form spheroids, demonstrating the impact of dynamic flow and static conditions to mimic cell damage and drug treatment. Liver inflammation was mimicked and verified using inflammatory markers. The study holds the potential for enhanced study of drug-induced liver injury (DILI) for drug screening.

## 2. Material and Methods

### 2.1. Fabrication of 24-Well Perfusable Spheroid Plate

**Figure 1:**
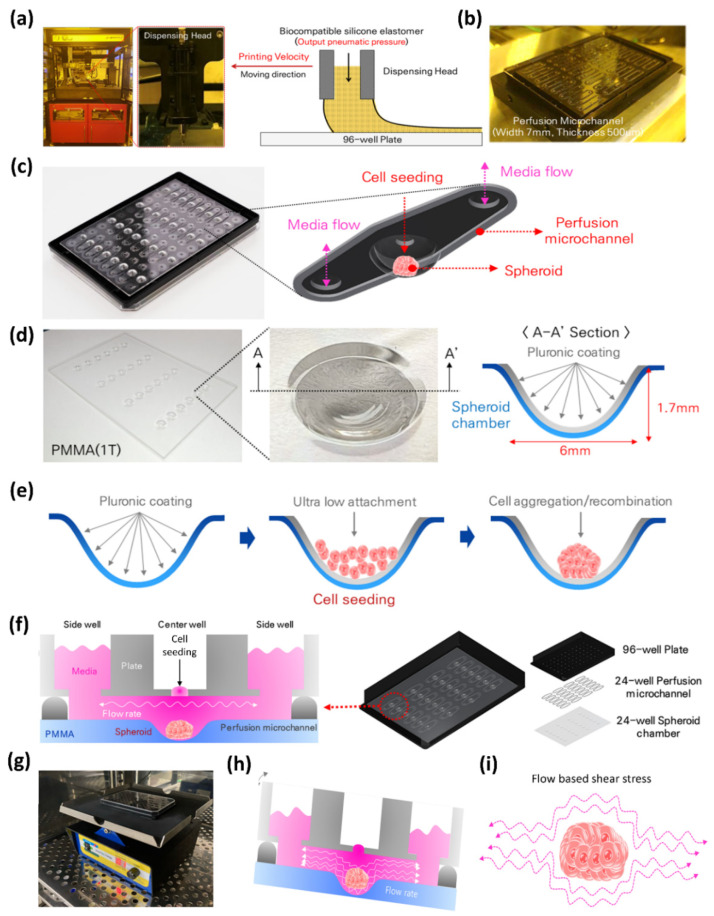
Plate fabrication and schematics of spheroid culture. (**a**) In-house build ink-jet printer for printing silicone to establish microfluidic channel across the array of 3 wells. (**b**) Printed microchannels for media perfusion. (**c**) Expanded view of microchannel. (**d**) PMMA layer with 24 well and spheroid well dimension. (**e**) Schematic of Pluronic127 coating, cell seeding, and spheroid formation. (**f**) Schematics of 3-well array for spheroid culture. (**g**) Mimetas™ rocker for dynamic spheroid culture. (**h**) Schematic of fluid flow in dynamic conditions. (**i**) Schematic of media perfusion inducing shear stress.

The spheroid culture plate, M-Physio™ 24-well perfusable spheroid plate (BioSpero Inc., Jeju, South Korea), was utilized in the current study. The fabrication process began with the black 96-well plate 33,396 (SPL Life Sciences, South Korea) for plate assembly. First of all, holes for media addition and cell seeding were drilled in the 96-well plate making arrays of three holes consisting of 2.5 mm diameter for side holes and a 1.4 mm diameter hole for the middle well. Hole processing was performed on a 96-well plate by using the commercially available numerical control (NC) equipment drilling system (Carvey^®^ 3D Carver, Inventables™, Chicago, IL, USA). After hole processing, the perfusion channels were printed on the backside of 96-well plate (Figure 1b). Biocompatible silicone elastomer (NuSil^®^, Avantor™, Carpinteria, CA, USA, Catalog # MED-6033) was selected for printing perfusion channel and an in-house built material jet 3D printing system (Figure 1a and Figure S1a,b) was used for elastomer printing on a 96-well plate. The 3D printing system dispensing process can control channel line width and thickness. Meanwhile, a PMMA (Polymethyl methacrylate) sheet was used for the fabrication of 24 wells for the spheroid culture, keeping the well positions at the central position of the perfusion channel (Figure 1d). A hemispherical well-shaped heating plate system, a spheroid chamber, was fabricated on a PMMA (width: 112 mm, length: 75 mm, T = 1 mm) sheet through a thermoforming manufacturing process at a temperature of about 130 °C. After the hole formation and perfusion channel printing on the 96-well plate and 24 hemispherical well formation on the PMMA sheet, both of them were assembled to develop the spheroid culture plate. Figure 1f shows the assembly pattern of plate development and the schematic of spheroid culture in the 3-well array system.

A PMMA-based hemispherical well-shaped cell culture chamber is functionalized to allow cells to aggregate through a Pluronic coating 1% solution (1 g of Pluronic127 powder in 100 mL of autoclaved water). Pluronic material has ultra-low attachment (ULA) properties. The 24-well dynamic spheroid plate consisted of 96 wells making 24 3-well arrays for perfusion microchannels. The array of three wells combines to form a perfusion microchannel for the media flow. In the three-well array, the middle well is comprised of a hemispherical hole for the spheroid chamber, and the two side wells were for media flow. Cell seeding is performed in the middle well, and media are supplied from the wells present on the sides of the middle well.

### 2.2. Cell Culture

Hepatocytes cell line HepG2 (HB-8065™, ATCC^®^, Gaithersburg, MD, USA), fibroblasts cell line HS68 (CRL-1635™ ATCC^®^, Gaithersburg, MD, USA), and primary human umbilical vein endothelial cells (HUVEC, C2519A, Lonza, Basel, Switzerland) were utilized in the current study. All the cell types were grown at 37 °C with 5% CO_2_ in a humidified incubator. All the cells were cultured for two passages before being utilized in experimental conditions. HepG2 and HS68 were cultured in DMEM with 10% FBS and 1% penicillin and streptomycin. Primary HUVEC cells were grown in endothelial cell media. The experiment for validation of the spheroid culture platform was divided into two phases. Initially, cell number was optimized for spheroid formation in a dynamic culture plate in which only hepatocytes (HepG2 cell line) were used. HepG2 cells were grown until confluency and then seeded in the spheroid plate at different concentrations of 2000, 5000, 10,000, 20,000, 25,000, and 30,000 cells for optimization of the cell number selection. The cells after seeding were progressively monitored for the spheroid formulation. The spheroids were observed in the inverted microscope to monitor their growth every 24 h. For dynamic culture, the spheroid plate was kept on a perfusion rocker (Mimetas B.V., The Netherlands) after the first 24 h of seeding the cells. 

### 2.3. Static and Dynamic Culture

For the feasibility of our dynamic culture spheroid plate, a comparative analysis of spheroids in static and dynamic culture was carried out for 3 days. Cells were grown in monoculture, both in the static and dynamic platforms. For static culture, the spheroids were grown without any rocker to mimic the traditional spheroid culture. The cells were seeded in the wells in numbers viz. 5000, 10,000, 20,000, 25,000, and 30,000 cells. The plates were kept in the incubator and monitored for seven days by observing the spheroid physiology. Mimetas Rocker was kept at the angle of 7° degrees and a rate of six rotation cycles per hour, maintaining the maximum stability for the spheroid culture in a dynamic environment.

### 2.4. Morphology Assessment of the Spheroids

The dimensions of the spheroids were measured by SpheroidSizer 1.0, an open-source software supported by MATLAB. The scale of the image was in microns per pixel (μm/pix). The dimensions included the volume (mm^3^), length (μm), and width (μm) of the spheroids. For the reconstruction of the 3D structure of the spheroids, the ReViSP tool (supported by MATLAB) was used.

### 2.5. Disease Modeling

Initially, spheroid formation was ensured in the dynamic culture system and the cell number was optimized at 20,000 cells per 20 μL. In dynamic conditions, disease modeling was performed to check the validity of the spheroid culture. For the disease modeling experiment, a mixed culture of three cell types was used for spheroid formation. A ratio of 5:4:1 for HepG2: HUVEC: HS68 was selected, and then cells were suspended in DMEM for cell seeding in the spheroid plate. For establishing a disease model, spheroids were cultured in normal media for the first 48 h. Subsequently, spheroids were exposed to pro-inflammatory cytokine interleukin 1-beta (IL-1β) (Sigma-Aldrich^®^, St. Louis, MO, USA, Catalog # SRP3083) after two days of normal growth. Spheroids were exposed to four different concentrations of IL-1β, i.e., 1, 5, 10, and 20 ng/mL for 5 days to cause cellular stress, leading to the inflammatory disease model. IL-1β was chosen to induce the cell damage in spheroids owing to its cell-damaging capacity. On the 7th day, the experiment was terminated, and immunostaining for ZO-1 (epithelial biomarker for hepatocytes) and CD31 (endothelial biomarker of HUVECs) was performed. The potential of the spheroid plate to model the disease in spheroid was analyzed by inducing cell injury by IL-1β with multiple aforementioned concentrations. Extracellular protein biomarkers were measured to analyze the dynamics of inflammation development at the maximum concentration of IL-1β (20 ng/mL), i.e., albumin, IL-6, and TNF-α. A normal spheroid culture was carried out as a reference for positive control without the exposure of IL-1β. 

### 2.6. ELISA

ELISA assays were performed for human albumin (ab108787, Abcam, Cambridge, MA USA), IL-6 (BMS213-2, Invitrogen, Seoul, South Korea), and human TNF-α (BMS223-4, Invitrogen, Seoul, South Korea) in accordance with the manufacturer’s protocol. Cell culture media were collected from the well at the given specific intervals. Culture media samples were centrifuged at 3000× *g* for 10 min and stored at −80 °C. The absorbance measurements were taken using SpectraMax i3 Multimode Microplate Reader (Molecular Devices, San Jose, CA, USA).

### 2.7. Brightfield and Immunofluorescence Microscopy

Light microscopy was performed to observe the spheroid formation after the intervals of 24 h, while cell optimization was performed at the end of Day 6 for assessment of static and dynamic conditions. Immunofluorescence staining was performed as an end-point assay after completing the 6-day experiment. For spheroid staining, hepatocyte biomarker ZO-1 and endothelial protein CD-31 were stained. 

### 2.8. Statistical Analysis

To validate the results, experiments were carried out in triplicate. To verify the statistical significance of data, a one-way analysis of variance (ANOVA) test was performed. Results are expressed as the means ± standard error of the means (sem) of three independent experiments. For statistical comparisons, a *p* value ≤ 0.05 was considered significant versus control and denoted by “*”, and *p* value ≤ 0.01 was considered highly significant versus control and denoted by “**”.

## 3. Results

### 3.1. Spheroid Characterizations and Cell Number Optimization

The current study elaborates the development of a perfusion-based microfluidic spheroid culture method. The PMMA sheet with 24 wells was fixed with a 96-well SPL Black Plate to develop a spheroid culture system. Figure 1a,b show the fabrication assembly of the spheroid culture plate and ink-jet printer system, which is further shown in Supplementary Figure S1. Figure 1c shows the expanded view of the 3-well array for the microfluidic culture of spheroids. Figure 1d elaborates the wells in a PMMA plate, and Figure 1e summarizes the process of cell aggregation for spheroid formation. Figure 1f–h shows the schematic view of the spheroid culture in a static method, the Mimetas™ rocker, which was used to create the perfusion of media and the schematic of a dynamic culture of spheroids, respectively.

**Figure 2:**
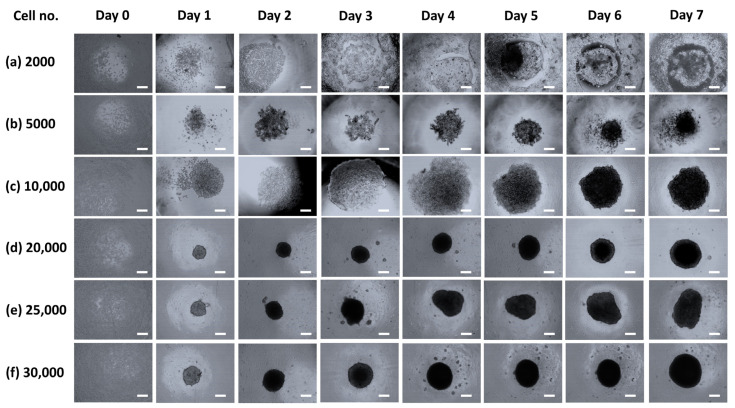
Representative images for cell number optimization; images were captured after each interval of 24 h until 168 h. (**a**) Growth pattern of 2000 cells indicated a failed spheroid formation, (**b**) 5000 cells showed cellular clumps until 96 h, which later transformed into spheroids. (**c**) 10,000 cells successfully indicated spheroid formation after 24 h in comparison with 2000 and 5000 cells. (**d**) 20,000 cell number was found to be the optimum cell number for proper spheroid formation. (**e**,**f**) 25,000 and 30,000 cell numbers exhibited spheroid formation similar to the cell concentration of 20,000 cells. (Scale: 100 μm).

The spheroids showed significant growth in the dynamic culture system. For the first 24 h after cell seeding, the plate was kept static for a spheroid formation and subsequently transferred to the rocker for dynamic culture conditions. Within 72 h, high density was observed in all the spheroids. For spheroid formation, the cell number was optimized out of six concentrations. The starting concentrations of spheroids with 20,000 and 25,000 cell numbers exhibited the sophisticated spheroid formation (Figure 2). Furthermore, albumin staining was assessed owing to its essential role in indicating hepatocyte functionality. To constitute a relevant hepatic in vitro system, cultured hepatocytes needed to be accurately formulated.

### 3.2. Comparative Analysis of Spheroids in Static and Dynamic Platforms

**Figure 3:**
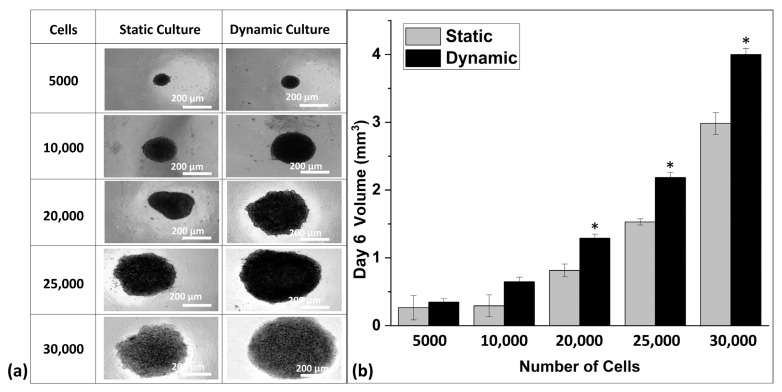
(**a**) Spheroid culture in static and dynamic culture; the spheroids at day 6. (**b**) Comparative volumetric analysis of spheroids in both static and dynamic culture. The spheroids in dynamic culture show large volumes as compared to their counterparts in static culture. * *p* ≤ 0.05 represents the statistically significant difference of spheroid size for dynamic versus static culture conditions.

After the optimization of the cell number for sophisticated spheroid formation, the efficiency of microfluidic perfusion of the microfluidic spheroid culture plate was ensured by a comparative culture of the spheroid in static and dynamic conditions. Spheroid formation and size were observed over the time of 3 days. Spheroid microscopic images (Figure 3a) indicated a significantly increased size of spheroids in dynamic conditions. Furthermore, the comparative graph of spheroid volume in Figure 3b indicated a clear difference with an increasing pattern of spheroid volume with a higher number of cells in dynamic culture conditions.

**Figure 4:**
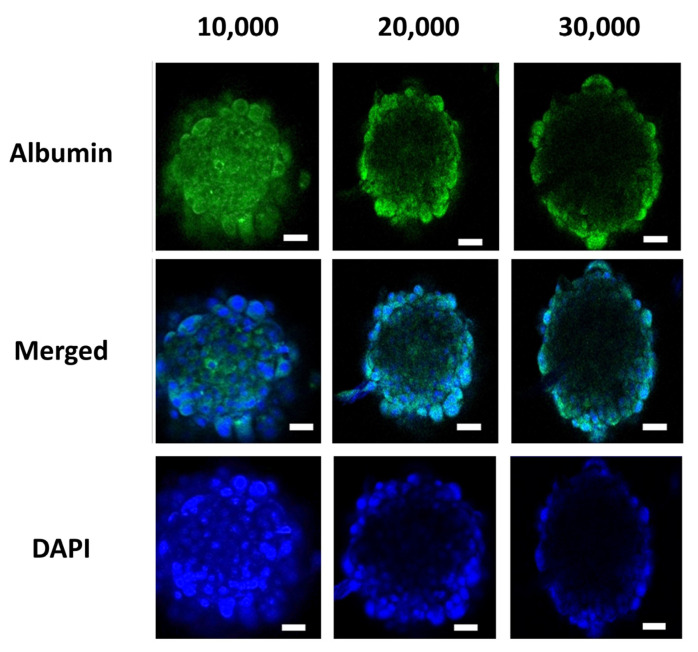
Representative images of the immunofluorescence staining of hepatocyte marker albumin (green) and DAPI (blue) at the end of Day 6. (Scale bar: 100 μm).

**Figure 5:**
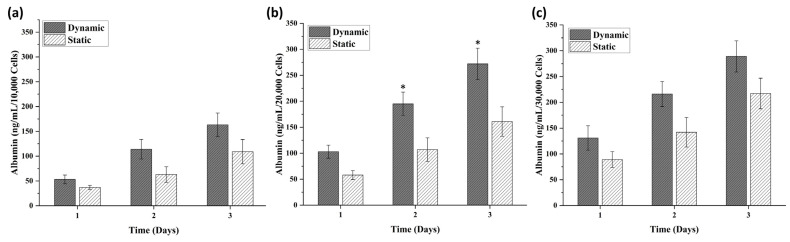
Static vs. dynamic culture albumin molecular biomarker analysis; (**a**) albumin secretion in spheroid with 10,000 cells; (**b**) albumin secretion in spheroid with 20,000 cells; (**c**) albumin secretion in spheroid with 30,000 cells. * *p* ≤ 0.05 for dynamic versus static culture conditions.

The volume of the spheroids was calculated based on the length and width of the spheroids measured by SpheroidSizer 1.0. As per the volumetric analysis, the spheroids grown in dynamic conditions were found to be larger in volume as compared to spheroids grown in static conditions (Figure 3b). This shows that, in dynamic perfusion-based spheroid culture, the spheroids grow in a significantly large size as compared to the static culture. The spheroids comprised of 5000 cells in both culture types showed no significant difference, but spheroids comprised of 10,000, 20,000, 25,000, and 30,000 cells showed a distinct difference in their volume with an increasing trend of size in dynamic culture conditions (Figure 3). To the optimized number of cells, the albumin staining was also performed, which showed the equal expression of albumin in the hepatic spheroids. Albumin immunofluorescent staining images revealed that spheroids with 10,000, 20,000, and 30,000 cell numbers showed albumin production after 6 days of culture (Figure 4). Additionally, extracellular expression of albumin protein was measured to analyze the comparative impact of cell number on its release. Figure 5b shows that albumin secretion in spheroids of 20,000 cells was found to be significant in comparison to spheroids with 10,000 and 30,000 cells, which did not indicate a significant difference.

### 3.3. Disease Modeling

**Figure 6:**
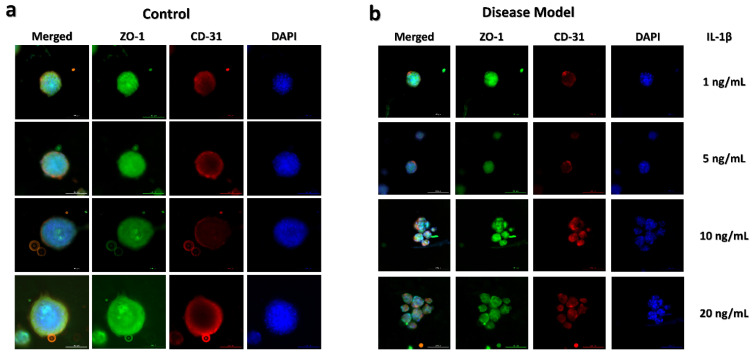
End-point expression of the ZO-1 tight junction and CD31 expression in disease model spheroids cultured in media with IL-1β (stimulant) versus control spheroids cultured with normal media (no stimulant) at Day 7. (**a**) Representative images of multi-staining results of 20,000 cell concentration for control samples in which spheroid formation has good ZO-1 expression in HepG2 cell line (epithelial cells) and CD31 expression in HUVECs (endothelial cells). (**b**) Representative images of disease model condition (IL-1β) treated spheroid showing different cluster formation but automatically overcome the inflammatory condition, spheroids were exposed to four different concentrations of IL-1β, i.e., 1, 5, 10, and 20 ng/mL for 5 days to cause cellular stress leading to the inflammatory disease model. ZO-1 is represented in green, CD31 is represented in red, blue staining represents nuclei (DAPI staining), and the three-color combination is a merged one. ZO-1 and CD 31 expression was comparatively lower than the control. (Scale bar: 200 μm).

**Figure 7:**
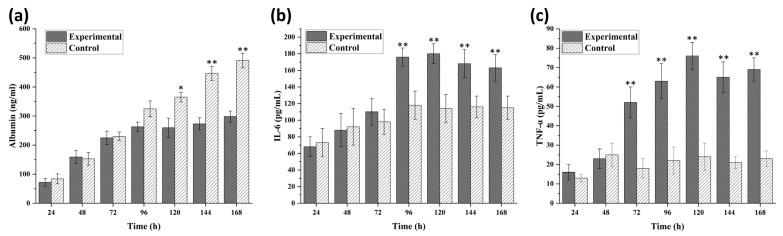
ELISA-based biomarkers quantification for disease model spheroids cultured in media with 20 ng/mL IL-1β (stimulant) versus control spheroids cultured with normal media (no stimulant) was performed after each interval of 24 h. Albumin (**a**), IL-6 (**b**), and TNF-α (**c**) were measured until one week to analyze the development of the inflammatory disease model under the influence of IL-1β. The results indicated the successful development of inflammation from Day 3. Data are shown as mean ± SEM. * *p* ≤ 0.05 versus control; ** *p* ≤ 0.01 versus control.

IL-1β, a pro-inflammatory protein, was selected to induce inflammation in liver spheroids [34]. The inflammation model development in the spheroids was performed with the exposure of IL-1β starting from Day 3. The monitoring of inflammation state in the spheroids was confirmed using albumin, IL-6, and TNF-α secretion at specific intervals and end-point spheroid staining with ZO-1 and CD-31 for observing spheroid shape (Figure 6 and Figure 7). Albumin, being a decisive biomarker for hepatocyte health, was selected for disease monitoring during the IL-1β exposure experiment. The disease modeling was performed with the spheroids formed at 20,000 cells per 20 μL of media at the starting point. Albumin ELISA results of the disease model further validated the dynamic culture growth after pro-inflammatory cytokine exposure. The results indicated that dynamic flow conditions for spheroid culture overcame the stress simultaneously, with an increased level of albumin production observed with the passage of the time in Figure 7a. No significant difference was found until the first 72 h of spheroid culture. Subsequent to IL-1β exposure, albumin concentration reduced until 120 h as compared to the control samples. From 120 h, media containing IL-1β were replaced with fresh normal media and samples taken at 144 and 168 h. In Figure 7b,c, an increased expression of IL-6 and TNF-α can be observed, respectively. For the validation of inflammation development, the samples for extracellular biomarker quantification were taken from the maximum concentration of IL-1β exposure, i.e., 20 ng/mL. 

## 4. Discussion

We have successfully developed a high-throughput dynamic plate-based microphysiological system, establishing spheroids to mimic the human microtissue environment. The microfluidic gravity-based perfusion system provided the continuous media flow across the periphery of the spheroid, overcoming the limitation of providing media exposure across the spheroid surface equally and continuously. The phenomenon of molecular gradient formation may also exist in static spheroid culture as one of its limitations, which has also been addressed in the current perfusion system, making a continuous molecular movement in media due to gravity-based perfusion. The plate consisted of 24 arrays of three wells for the culture system hosting 24 spheroid tissues. Supplementary Figure S2a shows the holes drilled in the microwell plate with diameters of 2.5 mm for side wells and 1.4 mm for the middle well. The smaller diameter of the middle well was used for cell seeding and kept open for the rest of the experiment, and only the 96-well plate cover was given. Based on the surface tension principle, the middle opening does not affect media flow, and the media did not flow out of it, making the perfusion possible across the side wells; a schematic of the image has also been given in the Supplementary Figure S2b. In Supplementary Figure S3a, an assembled macro view of the 3-well array with the printed silicon elastomer has been shown to have a smaller hole middle opening. The middle well was later coated with 1% Pluronic coating P127 to make the surface highly hydrophobic and resist cell attachment for spheroid formation (Supplementary Figure S3b). Pluronics or Poloxamers are non-toxic FDA-approved poly (ethylene oxide)/poly(propylene oxide)/poly(ethylene oxide) (PEO-PPO-PEO) triblock copolymers. An extensive literature is available for Pluronic F127 (F127), investigating its usage in cell culture and as a drug carrier owing to its biocompatibility, minimal toxicity properties, high drug-loading capabilities, and gel formation capacity in physiological conditions [35,36,37].

Continuous evidence has shown that among 3D culture models, spheroids and organoids have indicated relatively improved emulation of human microphysiology [38,39,40]. The perfusable platform for spheroid culture utilizing a dynamic plate showed a slightly significant proliferation and metabolic assay. Mainly 3D models focus on emulating one specific function of a tissue, whereas spheroid models present with the opportunity to mimic multiple functions of tissue based on the cell types, extracellular matrix, and scaffold size. Spheroid culture integrates multiple cell types into one suspended tissue form to increase the intercellular crosstalk. The currently studied plate was designed to enhance the physiological emulation of spheroid models by introducing the perfusable culture to mimic the in vivo fluid interaction of a tissue. The hepatic model was chosen for the study objective due to its crucial role as a metabolic hub for disease and drugs.

The fabricated microfluidic plate for the spheroid culture system presented with sophisticated spheroid models to mimic liver inflammation. Prior to disease modeling, a specific cell number for spheroid culture was optimized for static culture and dynamic culture. The plate displayed sustainable spheroid formation at a range of cell numbers from 10,000 cells per 20 μL to 30,000 cells per 20 μL of media. The selected cell number for disease modeling was optimized at 20,000 cells per 20 μL. The comparative studies between the static and dynamic culture of spheroids showed a good morphological manifestation of spheroid volume in dynamic culture as compared to the static culture. Previously, Baze et al., reported the spheroid culture of primary hepatocytes and non-parenchymal cells (2:1) for a long period of 14 days [41]. We co-cultured the triad of cells in the physiological ratio 5:4:1 of HepG2: HUVEC: HS68, for spheroid formation. This work is the beginning of 3D spheroid culture in a dynamic flow platform; thus, more emphasis is required, in the future, for the multi-cultured spheroids, comprised of iPSCs derived parenchymal and supporting non-parenchymal cells for DILI and other toxicity studies.

The key aspect of the formation of 3D culture systems is to mimic human physiology for improved disease modeling for drug screening. The disease modeling experiment was designed to emulate the inflammation of the liver spheroid. IL-1β induced inflammation was monitored in spheroid for 5 days in dynamic culture, which showed the reduced metabolism and spheroid size. Figure 6 shows the representative images of disease modeling, which may be validated by the albumin, IL-6, and TNF-α production pattern in the given spheroids (Figure 7). The significant increase in IL-6 and TNF-α release validated a continuous development of inflammation state in the spheroid model with a simultaneous decrease in albumin production from Day 3. The reduced spheroid size and albumin production indicate the induction of inflammation in the spheroids with a gradual decrease in the biomarker. At Day 7, the media containing IL-1β was removed, which led to a steady rise in the albumin concentration. However, the current study has analytical limitations such as cell viability assay, mRNA expression for disease confirmation biomarkers, and H&E staining of spheroid tissues. For media flow perfusion, the Mimetas Rocker was kept at the angle of 7° degrees and a rate of six rotation cycles per hour, maintaining the maximum stability for spheroid culture in a dynamic environment. Analysis of spheroid formation and growth at different angles and different media perfusion speeds is further required, which may impact different gene expression profiles. Additionally, the integration of sensors for continuous monitoring of the cellular microenvironment may enhance the disease modeling and drug screening capacity for such high-throughput platforms. 

The current platform with channel possesses the potential for a steady fluid flow across the spheroid surface, which is more relevant to fluidic flow in human microvasculature. A spheroid culture method with microfluidic capacity mimics the human microtissue environment relatively better as compared to a static spheroid platform. Additionally, continuous evidence has supported that organ-specific differential expression of genes and phenotype is better observed in dynamic spheroid culture platforms [42,43,44]. Although recent evolution in 3D cell culture models to mimic human physiology in vitro has improved MPSs capacity, there is a lack of vascularization and perfusion in spheroid cultures. The designed spheroid culture system tries to address the issue of culture media perfusion for equal distribution of nutrients across the spheroid surface. The spheroid culture system successfully indicated consistent results with spheroid size and growth for the optimized number of cells. The microfluidic perfusion plate provides a spheroid culture in dynamic conditions to overcome one of the main limitations of equal distribution of media as compared to conventional spheroid culture techniques. In conclusion, the designed plate may prove to be an efficient tool to model disease for drug screening at the initial stages of drug development.

## 5. Conclusions

The perfusion-based culture system holds increased relevance to model an inflammatory hepatic microenvironment as compared to the static spheroid culture. The findings validate that the perfusion-based method for a spheroid was found to be relatively effective in mimicking hepatic physiology as compared to the 2D cell culture technique. Perfusable media equips the plate with the circulation capacity that is more relevant to human physiology. The significant growth of spheroids as compared to the static culture platforms indicates that perfusion is a key factor in cell proliferation. The ultra-low attachment nature of our plate facilitated the growth of spheroids in suspension. The coculture of endothelial cells during spheroid formation and equal distribution of endothelial cells shows representation of endothelial cells in the hepatic spheroids. Furthermore, spheroid culture in dynamic flow has shown significantly relevant morphological manifestation as compared to the static culture. The spheroid model also emulated the hepatic microenvironment for disease modeling. The perfusable plate for spheroid culture enhances the capacity to mimic human organ physiology with increased relevance. 

## Data Availability

The data presented in this study are available on request from the corresponding author.

## Supplementary Materials

The following are available online at https://www.mdpi.com/article/10.3390/biomedicines9101369/s1, Figure S1: Inhouse built ink-jet printer., Figure S2: Schematics of the M-PhysioTM plate with dimensions., Figure S3: Macro view of perfusion channels and hemisphere well.
