# Supplementary material for: Gravity-Based Flow Efficient Perfusion Culture System for Spheroids Mimicking Liver Inflammation
Source: Biomedicines. 2021 Oct 1;9(10):1369. doi: 10.3390/biomedicines9101369 (PMC8533112; doi:10.3390/biomedicines9101369)
Supplement: Supplementary file 1 [file biomedicines-09-01369-s001.zip › biomedicines-1371359-supplementary.pdf]

Supplementary Material

Article

Gravity-based Flow Efficient Perfusion Culture System for Spheroids Mimicking Liver Inflammation

Young Su Kim [1,†], Arun Asif [2,†], Abdul Rahim Chethikkattuveli Salih [2], Jae Wook Lee [2], Ki Nam Hyun [1] and Kyung Hyun Choi [1,2,*]

[1] BioSpero Inc., Jeju-si 63243, Jeju–do, South Korea; youngsu1742@naver.com (Y.S.K.); gusrlska8204@naver.com (K.N.H.)
[2] Advanced Micro Mechatronics Lab., Department of Mechatronics Engineering, Jeju National University, Jeju-si 63243, Jeju-do, South Korea; arunasif@hotmail.com (A.A.); abdul.rahim350@gmail.com (A.R.C.S.); jaewook482@gmail.com (J.W.L.)
* Correspondence: amm@jejunu.ac.kr; Tel.: +82-64-754-3713; Fax: +82-64-752-3174
† Authors contributed equally.

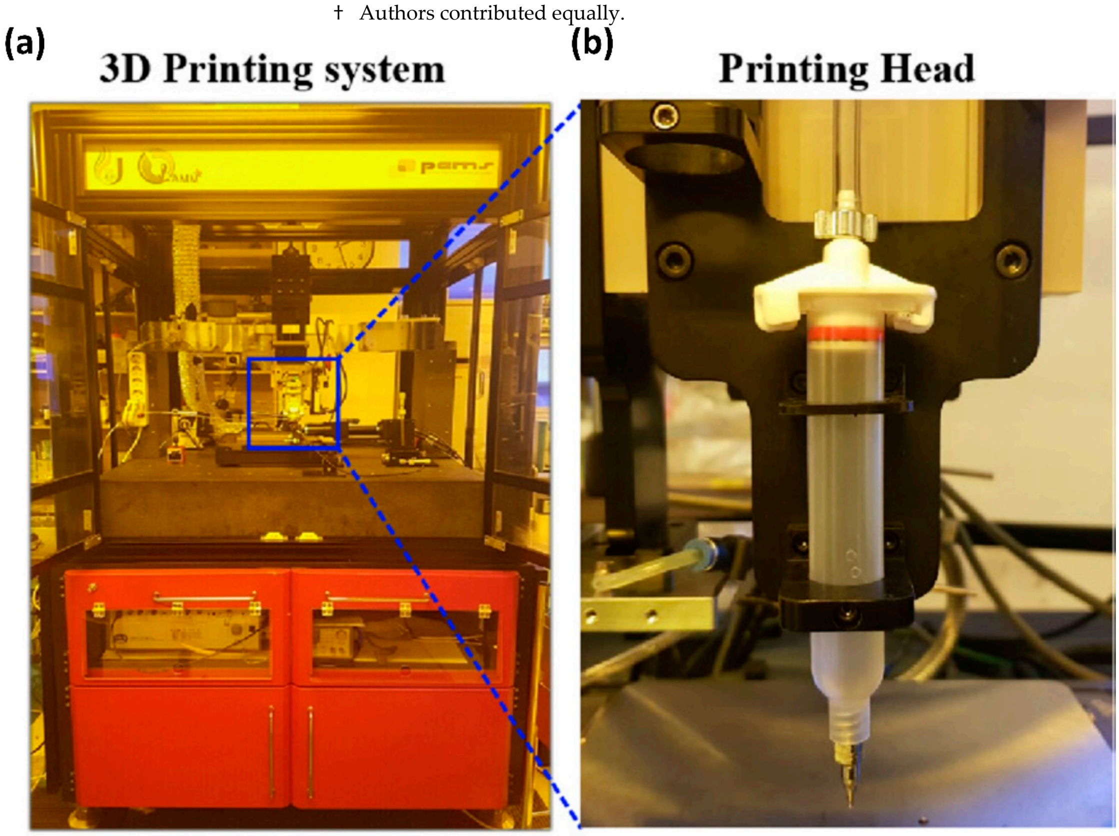

Figure S1. Inhouse built ink-jet printer. (a) EHD Multi-head system for 3D channel printing. (b) printing head.

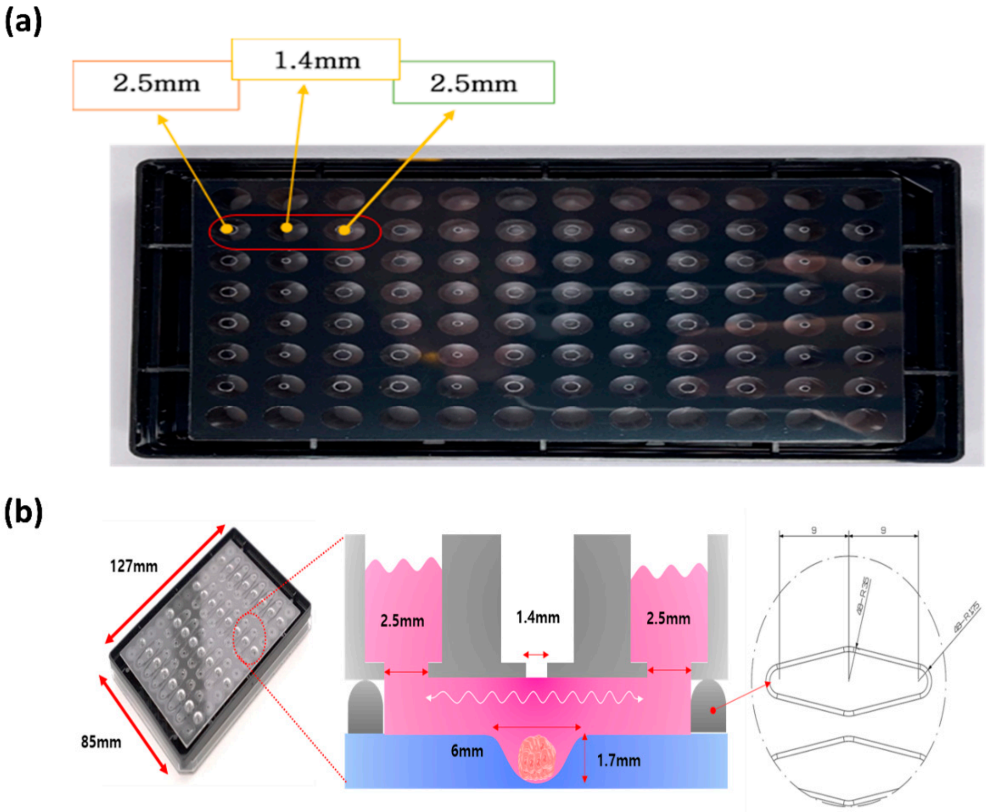

Figure S2. Schematics of the M-PhysioTM plate with dimensions (a) Black plate used to fabricate PMMA hemisphere spheroid wells in the bottom. (b) The schematics of the M-Physio^{TM} plate with dimensions.

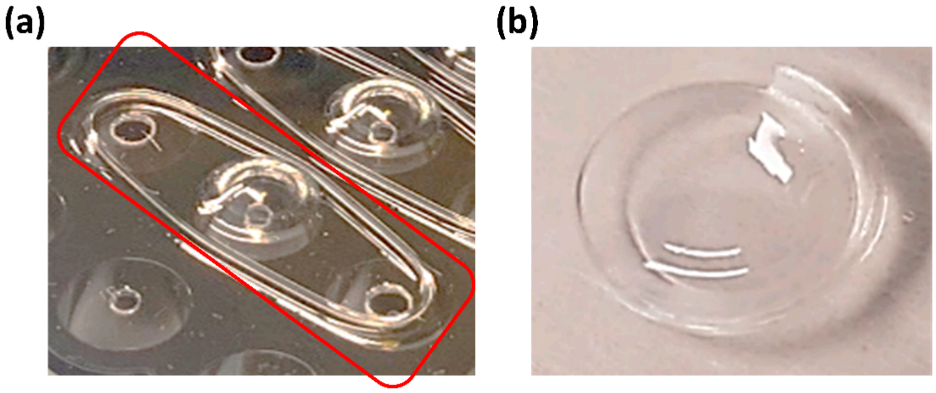

Figure S3. Macro view of perfusion channels and hemisphere well (a) The macro view of perfusion channels in the hemisphere wells (b) 1% Pluronic coating in the hemisphere well.
